# Editorial: The chemokine system in epithelial immunity

**DOI:** 10.3389/fimmu.2024.1481686

**Published:** 2024-08-29

**Authors:** Géraldine Schlecht-Louf, Sara Federici, Bernhard Ryffel, Remo Castro Russo

**Affiliations:** ^1^ INSERM U996 Inflammation, Chimiokines et Immunopathologie Châtenay-Malabry, Orsay, France; ^2^ Systems Immunology Department, Weizmann Institute of Science Rehovot, Rehovot, Israel; ^3^ Immuno-Neuro Modulation-INEM National Centre for Scientific Research (CNRS-UMR7355) and University of Orleans, Orleans, France; ^4^ Laboratory of Pulmonary Immunology and Mechanics, Federal University of Minas Gerais, Belo Horizonte, Minas Gerais, Brazil

**Keywords:** chemokine, COPD exacerbation, COVID-19, skin wound healing, Sjögren’s syndrome, autoimmunity

Chemokines create gradients that guide the directional migration of immune cells. By establishing these gradients within epithelial tissues, chemokines help in the precise positioning of immune cells, ensuring an effective immune response to threats while maintaining tissue homeostasis (1, 2). The Research Topic *“The Chemokine System in Epithelial Immunity”* elucidates how chemokines influence epithelial immunity in various contexts (healthy, infected, or inflamed tissues). This includes exploring how chemokines affect tissue-specific health and immune responses in mucosal sites such as lungs, skin, and secretory glands in the mouth and eyes.

Cytokines drive Chronic Obstructive Pulmonary Disease (COPD) exacerbations, leading to critical events that elevate mortality risk and also accelerate lung function decline (3). The inflammatory response during these exacerbations worsens airflow limitation, yet its effects on epithelial repair remain insufficiently understood. Addressing this gap, Kortekaas et al. explore how cytokines influence the lung microenvironment during COPD exacerbations and epithelial repair mechanisms. Using an exacerbation cocktail (EC) composed of IL-1β, IL-6, IL-8, and TNF-α (elevated during COPD exacerbation), the researchers examined epithelial progenitor behavior in mouse organoids and matured epithelial cell responses in precision-cut lung slices (PCLS). The findings revealed that EC exposure significantly enhances organoid formation and growth, with notable upregulation of *Lamp3*, *Muc5ac*, and *Muc5b* in organoids, indicating altered epithelial differentiation. Interestingly, while EC exposure did not affect epithelial marker expression in PCLS, pre-treatment of fibroblasts with EC increased organoid formation. This suggests that EC modifies the epithelial progenitor support function of fibroblasts, mainly through the expression of *Il33*, *Tgfa*, and *Areg*, although these factors alone did not replicate the observed effects on organoid formation. The study concludes that EC exposure stimulates organoid growth and disrupts epithelial differentiation, implicating the altered microenvironment in exacerbation-associated epithelial dysfunction. These findings provide important insights into the complex interplay between inflammation and epithelial repair in COPD, highlighting potential therapeutic targets to mitigate the detrimental effects of exacerbations on lung health.

Pulmonary diseases remain a leading cause of global mortality, with the COVID-19 pandemic underscoring the severe impact of viral infections on public health. While vaccines have reduced COVID-19-related deaths, managing severe cases marked by uncontrolled inflammation is still challenging. The chemokines are involved in the excessive recruitment of leukocytes into the lungs, particularly neutrophils, leading to significant tissue damage (2, 4). The recent research by Oliveira et al. highlights the role of glycosaminoglycan (GAG)-binding chemokine fragment CXCL9(74-103), which shows promise in modulating this inflammatory response. In two murine models of coronavirus-induced pneumonia—MHV-3 and SARS-CoV-2-the treatment with CXCL9(74-103) reduced leukocyte accumulation in the lungs, improved lung function, and decreased tissue damage. Moreover, in the SARS-CoV-2 model, CXCL9(74-103) also lowered viral titers, suggesting its potential to help viral clearance. These findings support CXCL9(74-103) as a promising therapeutic candidate for managing severe pulmonary viral infections by effectively reducing inflammation while promoting viral clearance. Further research is warranted to confirm these benefits in clinical settings, potentially offering a new approach to treating COVID-19 and similar diseases.

The chemokine SDF-1/CXCL12 plays a pivotal role in the migration and function of various precursor cells through its interaction with the receptor CXCR4 during skin wound healing (5). Despite the well-documented biological functions of SDF-1, its clinical applications remain underexplored. This gap is mainly due to the chemokine’s complex biology, including its systemic and local effects, reliance on amino-terminal processing, and the limited understanding of modulators that can enhance its stability and function. Recent findings presented by Pereira et al. highlight a novel approach to stabilizing SDF-1/CXCL12 using chlorite-oxidized oxyamylose (COAM), a macromolecular compound that protects SDF-1 from proteolytic degradation by inflammation-associated proteases, specifically MMP-9 and DPPIV/CD26. *In vitro* studies demonstrated that COAM preserves SDF-1 activity, maintaining its ability to mediate key cellular functions such as intracellular calcium mobilization, ERK1/2 phosphorylation, receptor internalization, and chemotaxis of CXCR4-positive cells. Moreover, *in vivo* experiments using a murine model showed that the combination of COAM and SDF-1, delivered via a fibrin hydrogel, significantly improved skin wound healing. These findings suggest that COAM-mediated protection of SDF-1 from proteolysis represents a promising therapeutic strategy to enhance SDF-1 bioavailability, with potential applications in wound healing and beyond.

Sjögren’s syndrome (SS) is a chronic autoimmune condition characterized by infiltrating lymphocytes, dendritic cells, and macrophages into exocrine glands, in which chemokines are critically involved (6). The mechanisms driving this inflammatory response involve a range of molecular interactions, including the crucial roles of lymphocyte homing receptors and their ligands, such as α4β7/MAdCAM-1, LFA-1/ICAM-1, CXCL13/CXCR5, CCL25/CCR9, and CX3CL1/CX3CR1. These interactions facilitate the migration of inflammatory cells to glandular tissues and contribute to the pathogenesis of SS. A recent review by Liao et al. discussed how various molecules, including TNF-α, IFN-α, IFN-β, and B cell activating factor (BAFF), are involved in lymphocyte homing. This process is regulated primarily through the Janus kinase-signal transducer and activator of transcription (JAK-STAT) pathway, the lymphotoxin-β receptor pathway, and nuclear factor-κB (NF-κB) signaling. Such insights have spurred the development of novel therapeutic strategies targeting these pathways, including antibodies against cell adhesion molecules, chemokine receptor antagonists, and gene therapies aimed at modulating chemokine signaling. The current review emphasizes the pivotal role of lymphocyte homing in SS pathogenesis and highlights the progress in targeted therapies. By understanding and intervening in the mechanisms of lymphocyte migration, these novel therapeutic approaches offer promising avenues for improving treatment outcomes in SS patients.

Collectively, cytokines and chemokines are pivotal in orchestrating immune responses and influencing disease outcomes across various conditions (1–6). Understanding their roles in COPD exacerbations, COVID-19, skin wound healing, and Sjögren’s Syndrome can lead to better diagnostic markers and more targeted therapies (3–6). Ongoing research into chemokine signaling pathways and their regulation will continue to provide insights into managing these complex diseases effectively, uncovering new mechanisms of immune regulation, and potentially identifying new targets for intervention. Thus, this topic depicted the advances in understanding immune regulation and developing novel therapeutic strategies regarding chemokines in epithelial immunity.
